# Th1/Th2 balance and humoral immune response to potential antigens as early diagnostic method of equine *Strongylus* nematode infection

**DOI:** 10.14202/vetworld.2017.679-687

**Published:** 2017-06-24

**Authors:** Faten A. M. Abo-Aziza, Seham H. M. Hendawy, Amira H. El Namaky, Heba M. Ashry

**Affiliations:** Department of Parasitology and Animal Diseases, National Research Centre, Cairo, Egypt

**Keywords:** cytokines, interleukin-4, *Strongylus*, Th1, Th2, tumor necrosis factor alpha

## Abstract

**Aim::**

The aim of this study was to investigate the early diagnosis of strongyle infection based on early changes in Th1 and Th2 cytokines beside the diagnostic accuracy values and sodium dodecyl sulfate-polyacrylamide gel electrophoresis (SDS-PAGE) and western blotting profiles using prepared strongyles antigens.

**Materials and Methods::**

A total of 73 donkeys had a mean age of 4-32 years old were parasitologically examined for strongyle infection. The early changes in Th1 and Th2 cytokines were determined, and the diagnostic accuracy values and SDS-PAGE and western blotting profiles were performed using prepared strongyles antigens; crude somatic *Strongylus* *vulgaris* (CSS), excretory-secretory *S. vulgaris* (ESS), crude somatic *Cyathostomins* (CSC), and excretory-secretory *Cyathostomins* (ESC).

**Results::**

The results revealed highest 437.04% and lowest 37.81% immunoglobulin G (IgG) in high and low egg shedder groups when using ESC and CSS antigens, respectively. Antibodies index for ESS and CSC were significantly higher in moderate egg shedder group while that for ESS and CSC, ESC was significantly higher in high egg shedder group. Tumor necrosis factor alpha (TNF-α)/interleukin-4 (IL-4) balance in *S. vulgaris* infected donkeys was approximately equal in apparently healthy, low and high egg shedder groups while TNF-α < IL-4 in moderate egg shedder. In *Cyathostomins* infected animals, TNF-α/IL-4 balance was approximately equal in apparently healthy group while it was low in moderate and high egg shedder groups. The diagnostic accuracy showed that the higher specificity (46.6%) and prevalence (95.40%) were recorded by CSS and ESC antigens, respectively. However, SDS-PAGE and western blotting profiling proved that the band at molecular weight 25 kDa is exhibited by CSS antigen.

**Conclusion::**

Combination of detecting level of TNF-α/IL-4 balance, CSS antigen and IgG concentration is good tool for appropriate diagnosis of such infection. More advancement research must be done concerning Th1/Th2 balance and cross-reactivity of *S. vulgaris* and *Cyathostomins* spp. at the base of serological and molecular investigation.

## Introduction

Equine strongylosis has long been distributed throughout the world [1]. It caused highly prevalent complex mixed infections of all ages [2]. It classified as *Strongylinae* as large and *Cyathostominae*, as small strongyles. Life cycle of both strongyles has two phases; free-living phase and parasitic phase. Eggs passed with fecal material where the 1^st^, 2^nd^, and 3^rd^ stage larvae developed. Climatic condition affects developing of the infective 3^rd^ stage larvae and its migration on water films and vegetation. When it ingested by the host, it passes to the small intestine and initiates the development of the parasitic phases. The large strongyle larvae molt into 4^th^ larval stages which migrate to cranial mesenteric arteries and molt into 5^th^ larval stages and complete their development into an adult at caecum and large intestine. However, small strongyles burrow in mucosa and submucosa where become encysted and molted into 4^th^ larval stages and reach their adult stage in the large intestine and colon [1]. The most pathogenic species of the large strongyles is *Strongylus vulgaris* [3]. Although the severity of adults in damaging the gut is of less consequence than those caused by the migrating larval stages that induce endoarteritis in the mesenteric artery and provoke thickening of the arterial wall resulting in verminous aneurysm [4,5]. Abdominal distress and colic are observed as the most common clinical symptoms. In foals, clinical signs such as a rise in temperature, anorexia, depression, and abdominal pain have been regarded [6]. Virtually, a great proportion of strongyle nematodes’ total burden existent in the gut lumen is *Cyathostomins* spp. which comprises 51 species in 13 genera. Its numbers usually vary from a few thousand to more than one million and distributed throughout the dorsal and ventral colon and occasionally caecum. All equids can harbor tens of thousands of these parasites without developing a clinical illness due to inhibited mucosal larvae embedded in the lining mucosa. Nonetheless, in some individuals, large numbers of inhibited mucosal larvae reactivate simultaneously causing a severe inflammatory colitis, associated with weight loss, diarrhea and subcutaneous edema and/or pyrexia [1,7].

Traditionally, the diagnosis of strongyle infection has based on conventional parasitological methods such as fecal egg count (FEC). It’s the commonly used routine for diagnosing strongyle infection but, do not reveal either the burden of sexually immature stages or distinguish strongyle eggs of different species [8]. Hence, larval cultures are compulsory for development and differentiation of larval stages. Based on morphological features diversity of the infective 3^rd^ stage larvae, strongyle nematodes can be identified to genus or species level [9]. The diagnosis of such infection is difficult as there are no pathognomonic clinical or hematological features [1]. The equine immune response to strongyle nematode is engaged with complexity of the helminths’ life cycle that involves numerous developmental stages. Further, arrival of adult in the gut lumen and penetration of its mucosa presents the host with multivariate antigenic challenge, immune stimulation, and immune modulation [1]. The most promising recent advance in this field has been involved interaction between helminths and immune cells of their hosts. Moreover, nematode infection expresses in two different ways. First, expulsion of nematodes from the intestines of host species occurs during primary infection; this phenomenon is known as self-cure and possibly involves innate immunity. Second, hosts which have become infected often show sterile immunity following resolution of the infection which probably involves adaptive immune responses, which may be primed by regular low exposure to nematodes on pasture [10]. Several proteins produced by strongyle nematode were involved in the regulation of T-helper (Th2) cytokines such as interleukin-4 (IL-4) production as well as, there is evidence that tumor necrosis factor alpha (TNF-α) is a key mediator involved in initiating an inflammatory cascade [7]. Furthermore, nematode infection-induced Th2 dominant immune responses that activate mucosal mast cell intestinal eosinophil and antibody class switching by plasma cells; immunoglobulin M (IgM), IgE, and IgG. Despite the Th2 immune response, these nematodes are often able to persist in the host for a long-time resultant a chronic infection [7]. Immune evasion may be occurred due to the production of Th2-driving dendritic cell that induced by excretory-secretory antigens of such nematodes. Despite significant advances in parasite immunology research, immune response to this strongyle nematode remain in thoughtful how precisely helminths interact with their hosts and tolerate the complex immune responses generated against them [11].

The aim of this study includes an immunoassay that incorporates measuring of Th1 (TNF-α) and Th2 (IL-4) cytokines as well as serum total IgG using different prepared *S. vulgaris* and *Cyathostomins* spp. antigens to be used as a diagnostic tool refers to the stage of infection.

## Materials and Methods

### Ethical approval

This experiment was conducted in accordance with the guidelines laid down by the International Animal Ethics Committee and in accordance with local laws and regulations.

### Animals and samples collection

A total of 73 donkeys had a mean age of 4-32 years old with different gender; 42 females and 31 males slaughtered at Giza zoo abattoir at the period from November 2015 to March 2016. At necropsy, large intestines, colons, and ceca were parasitologically examined for the existence of large and small strongyles spp. Donkey’s blood samples were collected into tubes containing no anticoagulant. After the clot was formed, the samples were centrifuged at 2000 rpm/10 min, and the serum was pipetted in small aliquots, which were stored at −20°C until analyzed for cytokines and total IgG. Individual rectal fecal samples were obtained and packed in plastic bags then, transferred to parasitology laboratory, where they were kept refrigerated until processed within 2 days.

### FEC

Fecal strongyle egg counts were analyzed quantitatively using a modified McMaster technique according to the RVC/FAO [12] guide to veterinary diagnostic parasitology, based on 4 g of feces and 56 ml floatation fluid. The McMaster counting chambers were filled with sieved stirred filtrate and allowed to stand for 5 min, then examined under compound microscope. FEC recorded as summation of a countable number of both chambers multiplied in 50 with a minimum detection level of 50 eggs per gram of feces (EPG). Eggs levels of FECs in all examined animals reflected the classification of animals into apparently healthy (0-49 EPG) and low (50-99 EPG), moderate (100-200 EPG), or high (>200 EPG) egg shedders. Each sample was counted for 3 succeeded times.

### Larval culture

Larval culture was performed according to the RVC/FAO [12] guide to veterinary diagnostic parasitology for fecal samples. In brief, 100 g samples of feces were weighed, put into plastic boxes, and incubated for 2-3 weeks at room temperature. During this time, the samples were regularly checked for desiccation, moistened if necessary, and ventilated for 1 h every day. After incubation, the 3^rd^ stage larvae (L3) were harvested by baermannization for 24 h. An aliquot of 100 µl was obtained from the sediment containing the accumulated larvae. All L3 of small and large strongyles were stained, immobilized and taxonomically identified according to Cernea *et al*. [13]. All cultures were examined by the same person.

### Parasites and antigen preparation

At necropsy, large and small strongyle worms were collected from the large colons and caeca of naturally infected donkeys. Collected worms were classified by morphological criteria into *S. vulgaris* and *Cyathostomins* spp. [13]. The identified worms were separately washed several times in phosphate buffer saline (PBS), pH 7.2 to remove any debris then, divided *S. vulgaris* and *Cyathostomins* spp. each into two batches, one for preparation of crude somatic antigen and the other for excretory-secretory antigen. Crude somatic *S. vulgaris* (CSS) and crude somatic *Cyathostomins* spp. (CSC) antigens were prepared [14]. Concisely, washed worms were homogenized in PBS pH 7.2 and centrifuged at 13,000 rpm/30 min in a cooling centrifuge. The supernatant was aspirated, and aliquots of each antigen were stored at −20°C until used. Excretory-secretory *S. vulgaris* (ESS) and excretory-secretory *Cyathostomins* spp. (ESC) antigens obtained with some modifications [5]. Briefly, living washed adult worms were incubated in 6 and 4 ml PBS pH 7.2 with penicillin (100 IU/mL), and streptomycin (100 IU/mL) for preparation of ESS and ESC, respectively, at 37°C/24 h in a 5% CO_2_ incubator. The buffer was collected then centrifuged at 10,000 rpm/20 min in cooling centrifuge. The ES antigens were dialyzed in dialysis bag with cut off 3.5 kDa. The dialyzed ES antigens were frozen at −20°C until used. Total protein content of all prepared antigens; CSS, CSC, ESS, and ESC were estimated [15].

### IgG analysis

Indirect enzyme-linked immunosorbent assay (ELISA) was optimized by serial checkerboard titration to the following setup. Optimal concentration of antigen, antibody and conjugate dilutions were chosen. 96 well microtiter plates (Grainger, Germany) were individually coated with 100 µl/well of each diluted antigen at the concentration of 4 and 6 µg/well for CSS, CSC and ESS, ESC, respectively, in carbonate-bicarbonate buffer, pH 9.6 and incubated at 37°C/1 h then, overnight at 4°C. After washing, the plates were blocked with 200 µl/well of blocking solution (2% dry skimmed milk in PBS - 0.05% Tween 20) and incubated at 37°C/1 h. The wells were washed then, 100 µl/well of diluted serum sample at 1:200 added to individual wells in duplicates and incubated for 2 h/37°C. Positive, negative, and blank controls were included on each plate in duplicates. The wells were washed 5 times with PBS Tween-20 (PBST), 100 µl/well of horseradish peroxidase (HRP)-conjugated goat anti-horse IgG conjugate diluted at 1:2500 were added and incubated for 1 h at 37°C. The plates were washed 5 times with PBST and incubated with 100 µl/well of substrate solution (O, phenylenediamine, 20 mg dissolved in 50 ml substrate buffer, pH 5 and 25 µl 30% H_2_O_2_) for 10-20 min at 37°C. The reactions were stopped with 100 µl of stopping solution (5% SDS) to each well and the optical densities’ (OD) at 450 nm were determined using an ELISA reader (BIO-TEK, Inc., ELx, 800 UV). The cut-off value based on measuring antibodies percentages and 20% of the positive control serum was selected to discriminate between positive and negative tested samples [16]. Diagnostic accuracy such as sensitivity, specificity, positive predictive value, negative predictive value, and apparent prevalence was calculated for different prepared antigens based on ELISA results in contrast to the parasitological investigation of the examined animals as following [16,17].


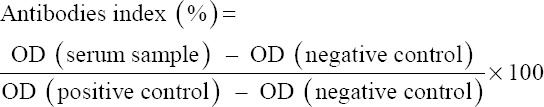



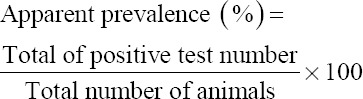



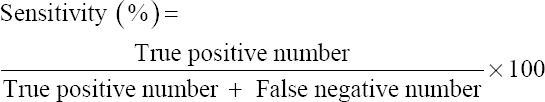



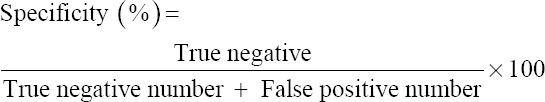



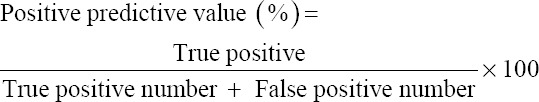



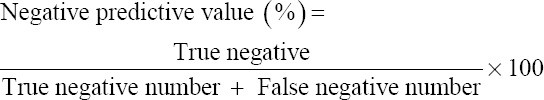


### TNF-α and IL-4 measurement

TNF-α and IL-4 concentrations in serum samples were measured by an equine-specific ELISA kits (WKEA MED Supplies) according to the manufacturer’s instructions. The manufacturer’s instructions were followed using purified TNF-α and IL-4 antibodies. The color change was measured spectrophotometrically at a wavelength of 450 nm, and the concentrations of TNF-α and IL-4 in the samples were determined by comparing the OD of the samples to the standard curve.

### Gel electrophoresis and western blotting

Both crude somatic and excretory-secretory antigens; CSS, CSC, ESS, and ESC were individually mixed with sodium dodecyl sulfate-polyacrylamide gel electrophoresis (SDS-PAGE) sample buffer and resolved in three 10% polyacrylamide gels under reducing conditions using pre-stained molecular weights protein marker (Fermentas and genedirex, USA) according to Laemmli [18]. After electrophoresis, one gel was stained with Coomassie Brilliant Blue R-250 dye and the others were transferred to 0.45 nitrocellulose membranes according to Towbin *et al*. [19]. Concisely, membranes were blocked for 1 h in 1% dry skimmed milk in PBS pH 7.2, then probed overnight with control positive naturally infected and control negative sera at 1:100 in Tris-buffered saline (TBS) with 0.5% bovine serum albumin (BSA) against all prepared antigens. The nitrocellulose strips were incubated with HRP-conjugated goat anti-horse IgG conjugate at 1:2500 in 0.5% BSA/TBS buffer for 1 h. The immune reactive bands were developed by incubation of the blot in the substrate solution (1-chloronaphthol [Sigma-Aldrich, USA], one tablet [30 mg/1 ml methanol] added to 10 ml methanol, 39 ml TBS and 30 µl 30% H_2_O_2_) for 5-10 min.

### Statistical analysis

The data were analyzed using Chi-square test by GraphPad Prism version 7 to compare the immunodiagnostic and accuracy values of antibodies index of different prepared *strongylus* antigens. Analysis of variance was used to compare blood levels of TNF-α and IL-4. Data were expressed as mean with standard error.

## Results

### FEC and larval culture

FEC data revealed that most animals were considered high egg shedder (69.86%) to strongyle eggs, followed by 13.69% for moderate egg shedder group, and 9.59% for apparently healthy group, then low egg shedder group (6.84%) (Table-1).

**Table-1:** Animal profile according to their FEC.

Total number of animals	n (%)			

	Apparently healthy 0-49 EPG	Low shedder 50-99 EPG	Moderate shedder 100-200 EPG	High shedder>200 EPG
73	7 (9.59)	5 (6.84)	10 (13.69)	51 (69.86)

Statistically significant (p<0.05). EPG=Eggs per gram, FEC=Fecal egg count

Larval culture assigned the actual parasitological existence of *S. vulgaris* and *Cyathostomins* spp. in all collected samples. The percentages were 75.86% and 86.21% for *S. vulgaris* and *Cyathostomins* spp., respectively.

### IgG analysis

ELISA results indicated that OD values of detecting antibodies (IgG) of either large and small strongyles were highly varied among different collected serum samples in their reactivity with *S. vulgaris* and *Cyathostomins* spp. prepared antigens; CSS, ESS and CSC, ESC, respectively. Results revealed that high egg shedder group was significantly higher (p<0.01) than that of apparently healthy group. However, moderate egg shedder group showed significantly higher with CSC antigen (p<0.05) and ESC antigen (p<0.01) than apparently healthy group. Among each group, ESC antigen was significantly higher than CSC antigen in the apparently healthy, moderate and high egg shedder groups at p<0.05, p<0.01, respectively (Table-2).

**Table-2:** OD values of IgG for CSS, ESS, CSC and ESC in donkey (450 nm).

Animals	CSS	ESS	CSC	ESC
Apparently healthy 0-49 EPG	0.423±0.053	0.559±0.018	0.538±0.017^a^	0.621±0.016^a^
Low shedder 50-99 EPG	0.462±0.016	0.537±0.022	0.511±0.023	0.608±0.056
Moderate shedder 100-200 EPG	0.561±0.019	0.562±0.033	0.655±0.025*^b^	0.783±0.035**^b^
High shedder>200 EPG	0.639±0.016**	0.679±0.012**	0.679±0.012**^b^	0.745±0.010**^b^

*, ** values were differed significantly from the value apparently healthy at p<0.05 and p<0.01, respectively. In the same group, a and b values within the same spp. are significantly different at p<0.05 and p<0.01, respectively. OD=Optical density, EPG=Eggs per gram, CSS=Crude somatic *Strongylus vulgaris*, ESS=Excretory secretory *Strongylus vulgaris*, CSC=Crude somatic *Cyathostomins,* ESC: Excretory-secretory *Cyathostomins* spp., and IgG=Immunoglobulin

The highest (437.04%) and lowest (38.73%) percentages of IgG occurred in high egg shedder group when using ESC and CSS antigens, respectively. Furthermore, moderate egg shedder group comprises highest (245.21%) percentage when using CSC antigen. As well as, low egg shedder and apparent healthy group include the highest percentage 238.1% and 293.2% while using ESC, respectively (Table-3). Comparing the antibodies index for CSS, ESS and CSC, ESC antigens in apparently healthy group it was found that antibodies index for ESS (p<0.01) and CSC (p<0.001) was significantly higher in moderate egg shedder group while that for ESS (p<0.01) and CSC, ESC (p<0.001) was significantly higher in high egg shedder group. Within each group, the results revealed that in apparently healthy and low egg shedder groups antibodies index for ESS antigen was significantly higher than CSS antigen (p<0.01) while, ESC antigen was significantly higher than CSC antigen (p<0.001) (Table-3). However, in moderate and high egg shedder groups antibodies index for ESS antigen was significantly higher than CSS antigen at p<0.01 and p<0.001, respectively.

**Table-3:** Immunodiagnostic values of antibodies index for CS and ES of *S. vulgaris* and *Cyathostomins* spp. in donkey (%).

Animals	Antibodies index (%)			

	*S. vulgaris* (large spp.)		*Cyathostomins* spp. (small spp.)	

	CSS	ESS	CSC	ESC
Apparently healthy 0-49 EPG	43.80±6.60^a^	158.6±18.10^a^	172.4±12.90^b^	293.2±13.70^b^
Low shedder 50-99 EPG	37.81±1.85^a^	210.0±13.00^a^	170.4±22.50^b^	238.1±24.20^b^
Moderate shedder 100-200 EPG	38.03±2.65^a^	282.4±17.90 **^a^	245.21±8.17***	232.1±13.00
High shedder>200 EPG	38.73±1.95^a^	240.9±10.60**^a^	373.1±27.40***	437.04±8.99***

Statistically significant (p<0.05). *,

** ,

*** values were differed significantly from the value apparently healthy at p<0.05 and p<0.01 and p<0.001, respectively. In the same group, values a and b within the same spp. are significantly different at p<0.01 and p<0.001, respectively. EPG=Eggs per gram, CSS=Crude somatic *Strongylus vulgaris*, ESS=Excretory secretory *Strongylus vulgaris*, CSC=Crude somatic *Cyathostomins*, ESC: Excretory-secretory *Cyathostomins* spp., and *S. vulgaris=Strongylus vulgaris*

Moreover, the diagnostic accuracy of the used antigens was elucidated by ELISA. It showed that the apparent prevalence of *Strongylus* infection was the highest using ESC antigen (95.40%) but, the CSS antigen noted the lowest prevalence (54%) (p<0.05). However, the highest sensitivity and positive predictive value percentages were recorded by ESC and CSS antigens 93.3% and 52.9%, respectively, p<0.05 while, specificity and negative predictive value percentages were 0% by ESC and CSC antigens. However, the lowest sensitivity (60%) was recorded by CSS antigen while the lowest positive predictive value percentage (45.8%) was showed when using ESS antigen (p<0.05) (Table-4).

**Table-4:** Diagnostic accuracy values using different prepared *S. vulgaris* and *Cyathostomins* spp. antigens by ELISA.

Parameters	Antigens				Chisquare

	*S. vulgaris*		*Cyathostomins* spp.		

	CSS	ESS	CSC	ESC	
Apparent prevalence %	54.00	80.20	89.60	95.40	p<0.05
Sensitivity %	60	73.3	86.6	93.3	p<0.05
Specificity %	46.6	13.3	0.00	0.00	-
Positive predictive value %	52.9	45.8	46.4	48.2	p<0.05
Negative predictive value %	53.8	33.3	0.00	0.00	-
Chisquare	p<0.05		p<0.05		-

Statistically significant (p<0.05). CSS=Crude somatic *Strongylus vulgaris,* ESS=Excretory secretory *Strongylus vulgaris,* CSC=Crude somatic *Cyathostomins,* *S. vulgaris=Strongylus vulgaris*, ESC: Excretory-secretory *Cyathostomins* spp., and ELISA=Enzymelinked immunosorbent assay

### Cytokines analysis and Th1/Th2 balance

Table-5 showed blood levels of TNF-α and IL-4 in apparently healthy and strongyle infected donkeys. Data revealed that both TNF-α and IL-4 blood levels in *Cyathostomins* infected moderate and high shedder groups were significantly higher than that in apparently healthy group. In *S. vulgaris* infected animals, IL-4 blood level was significantly higher than TNF-α blood level in moderate egg shedder group (p<0.05). TNF-α/IL-4 balance in *S. vulgaris* infected animals was approximately equal in apparently healthy, low and high egg shedder groups while TNF-α/IL-4 was low in moderate egg shedder group. As well as, in *Cyathostomins* infected animals IL-4 blood level was significantly higher than TNF-α in low, moderate (p<0.05), and high (p<0.01) egg shedder groups. TNF-α/IL-4 balance in *Cyathostomins* infected animals was approximately equal in apparently healthy group while it was low in moderate and high egg shedder groups.

**Table-5:** Blood levels of TNFa and IL4 in apparently healthy and strongylus infected donkeys (ng/ml).

Animals	*S. vulgaris* (large spp.)			*Cyathostomins* spp. (small spp.)		
	
	TNF-α	IL-4	TNF-α/IL-4 balance	TNF-α	IL-4	TNF-α/IL-4 balance
Apparently healthy 0-49 EPG	9.354±0.227	8.184±0.409	TNF-α≈IL-4	9.134±0.134	9.648±0.210	TNF-α ≈ IL-4
Low shedder 50-99 EPG	12.292±0.384	11.156±0.470	TNF-α≈IL-4	9.788^a^±0.276	13.168^a^±0.352	TNF-α<IL-4
Moderate shedder 100-200 EPG	14.742^a^±0.491	18.746^a^±0.237	TNF-α<IL-4	15.790*^a^±0.138	18.324*^a^±0.299	TNF-α<IL-4
High shedder>200 EPG	11.698±0.444	10.636±0.308	TNF-α≈IL-4	15.500*^a^±0.407	19.800**^a^±0.271	TNF-α<IL-4

*, ** values were differed significantly from the value apparently healthy at p<0.05 and p<0.01, respectively. In the same group, values with (a) within the same spp. are significantly different at p<0.05. TNF-α=Tumor necrosis factor alpha, IL-4=Interleukin-4, and EPG=Eggs per gram

### Electrophoretic and immune-reactive protein profile

**Figure-1:**
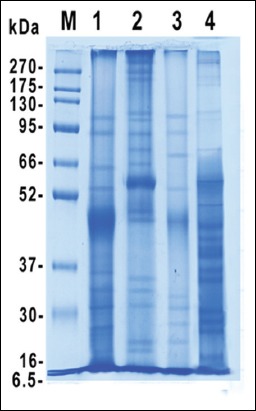
Electrophoretic protein profile of excretory-secretory *Cyathostomins* spp. antigen (Lane 1), excretory-secretory *Strongylus vulgaris* antigen (Lane 2), crude somatic *Cyathostomins* spp. antigen (Lane 3), crude somatic *S. vulgaris* antigen (Lane 4), and BLUltra pre-stained protein ladder, genedirex (Lane M, 6.5-270 kDa).

**Figure-2:**
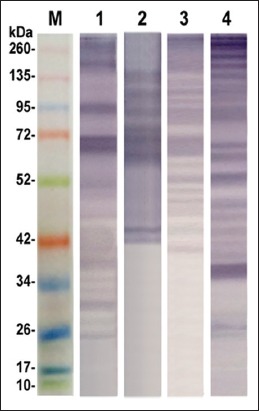
Immunogenic reactivity profile of excretory-secretory *Cyathostomins* spp. antigen (Lane 1), excretory-secretory *Strongylus vulgaris* antigen (Lane 2), crude somatic *Cyathostomins* spp. antigen (Lane 3), crude somatic *S. vulgaris* antigen (Lane 4) against control positive sera and pre-stained molecular weight protein ladder, Fermentas (Lane M, 10-260 kDa).

**Figure-3:**
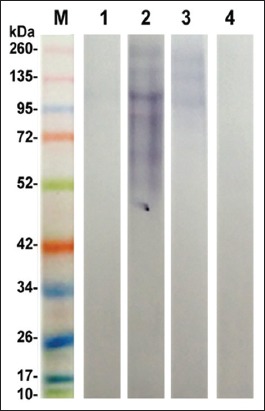
Immunogenic reactivity profile of excretory-secretory *Cyathostomins* spp. antigen (Lane 1), excretory-secretory *Strongylus vulgaris* antigen (Lane 2), crude somatic *Cyathostomins spp* antigen (Lane 3), crude somatic *S. vulgaris* antigen (Lane 4) against control negative sera and pre-stained molecular weight protein ladder, Fermentas (Lane M, 10-260 kDa).

SDS-PAGE is performed for resolving the protein bands in the prepared antigens; ESC, ESS, CSC, and CSS. The resultant data revealed 8 shared protein bands at molecular weights 22, 27, 31, 33, 46, 55, 92, and 108 KDa between ESC and CSC antigens. Furthermore, ESS and CSS shared in 9 common protein bands at the molecular weights 20, 35, 45, 48, 57, 76, 105, 113, and 270 KDa. Overall polypeptide bands exhibited by the different prepared antigens; ESC, ESS, CSC, and CSS were 11, 16, 9, and 18 protein bands at molecular weights 108, 92, 64, 55, 46, 36, 33, 31, 27, 22, and 19; 270, 264, 217, 113, 105, 90, 76, 67, 57, 48, 45, 35, 33, 31, 22, and 20; 108, 92, 72, 55, 46, 33, 31, 27, and 22, and 270, 235, 198, 113, 105, 76, 57, 48, 45, 42, 40,39, 36, 35, 30, 27, 25, and 20, respectively (Figure-1). Furthermore, the reactive profile of immunogenic bands displayed when such antigens verified against naturally infected and non-infected sera. The shared immune reactive polypeptides were detected at molecular weights 46, 50, and 260 KDa and 42 and 62 KDa in between ESC and CSC and ESS and CSS, respectively (Figure-2). The reactive band profiles presented by binding of specific IgG in positive naturally collected sera against each antigen; ESC, ESS, CSC, and CSS were identified at molecular weights 260, 181, 93, 69, 66, 50, 46, 38, 36, 33, 31, and 27 kDa, 137, 108, 90, 75, 68, 62, 46, 43, and 42 kDa, 260, 185, 113, 87, 67, 53, 50, 48, 46, 44, and 40 kDa, and 260, 224, 178, 139, 114, 87, 66, 62, 53, 48, 45, 42, 35, 27, and 25 kDa, respectively (Figure-2). On the other hand, the negative collected sera tested against such antigens revealed reactive bands at molecular weights 260, 176, 120, 94, and 71 kDa and 260, 177, and 127 kDa with ESS and CSC, respectively (Figure-3).

## Discussion

Early diagnosis of prepatent and patent stages of such strongyle infection is essential for the establishment of management and control strategy [20,21]. In this study, as the first step for appropriate diagnosis of strongyle infection, the animals were acknowledged to four different groups according to their FEC. Most animals were considered high egg shedder (69.86%), while (13.69%) were found to be moderate egg shedder and 9.59% and 6.84% were apparently healthy and low egg shedder, respectively. This finding has not reflected the existence of adult worms at necropsies where the animals may be under control of deworming protocol program or the primary host’s immune response that induce eviction of such strongyle nematode [7]. The main disadvantage of FECs is that they do not reflect the burden of sexually immature stages and also the eggs are not visible to the naked eye and special techniques together with a microscope are needed to detect their presence [22]. In this study, different prepared antigens of *S. vulgaris* (CSS, ESS) and *Cyathostomins* spp. (CSC, ESC) were evaluated to choose the promising antigen that could be used in early diagnosis of strongyle infection along with the immunoassay that incorporated the measuring of Th1 (TNF-α) and Th2 (IL-4) cytokines to be used as a diagnostic tool refers to the stage of infection.

Indirect ELISA results recorded highest apparent prevalence and sensitivity percentages when using ESC and CSC antigens. This finding may be attributed to the presence of immunogenic common bands at molecular weight 46, 50, and 260 kDa on blotting which could be responsible for detecting a high number of true positive in sera of naturally infected animals. In addition, variants species of *Cyathostomins* that can be existed within the same animal which nearly classified into about 13 genera comprises 51 species [13]. Furthermore, consequences of antigenic variation of *Cyathostomins* spp., host immune response did not exert differential effects of natural selection on those variants [1].

The ESC and CSC antigens lost its specificity and negative predictive value. This finding could be attributed to failure of these antigens in detection of true negative animal cases in parasitologically non-infected animals’ sera. In addition, these antigens scored high antibody index percentages even in low egg shedder and apparently healthy groups where these animals may be under control of deworming protocol program, inhibited mucosal larvae embedded in the lining mucosa or the primary host’s immune response that induce eviction of such strongyle nematode [1,23]. Likewise, ESS antigen possesses the shared immune reactive band at molecular weight 46 kDa which presented by ESC and CSC antigens in western blotting analysis. This band might be responsible for high antibody index, apparent prevalence and sensitivity percentages of ESS antigen. Interestingly, the CSS antigen showed the highest percentage of specificity 46.6% among different prepared antigens and succeeded in detecting the highest number of true negative animal cases in parasitologically non-infected animals’ sera. This result could be returned to the specific immunogenic band at molecular weight 25 kDa which exhibited by CSS antigen only in both SDS-PAGE and western blot profiles. This finding indicates the ability of this protein fraction to be presented by the host-parasite immune response and inducing formation of IgG against CSS antigen [10]. In addition, the lowest prevalence recorded by CSS antigen was 54% and sensitivity percentage 60%. This result could be attributed to this specific epitope which can recognize *S. vulgaris* species and does not cross react with other strongyles spp. [1]. A proportional relationship between antibodies and infection was found, but for appropriate diagnosis, TNF-α (Th1) and IL-4 (Th2) were measured. Elevation of TNF-α indicated a cellular immune response while increasing of IL-4 gave an idea of immune response shifting toward antibodies production [24]. In this study, both TNF-α and IL-4 blood levels in *Cyathostomins* infected moderate, and high egg shedder groups were significantly higher than that in the apparently healthy group, a picture of increased both immune arms. In contrast, IL-4 blood level in *S. vulgaris* infected animals was significantly higher than TNF-α blood level in moderate egg shedder group that might indicate weak infection that unable to stimulate the cellular response, but this infection could produce humeral response reflected by IL-4 elevation. These results might be attributed to the polarization of the immune response toward Th2 cytokines by helminths downregulated or altered protective Th1 cytokine responses [24]. In general, gastrointestinal nematodes are linked to the Th2-type lymphocyte response, including secretion of the following cytokines: IL-4, IL-5, and IL-13 [25].

Th1 and Th2 cytokines orchestrate different immune pathways to fight *Strongylus*; Th1 cytokines coordinate cellular immune responses, and Th2 cytokines coordinate humoral immune responses [26]. As well as, helminths infections are usually associated with polarized Th2/Th1 immune responses [27]. In this study, it was found that TNF-α/IL-4 balance in *S. vulgaris* infected animals was equal in the apparently healthy, low and high egg shedder groups while it was directed toward IL-4 elevation in moderate egg shedder group. However, TNF-α/IL-4 balance in *Cyathostomins* infected animals was equal in the apparently healthy while it was directed toward IL-4 elevation in low, moderate and high egg shedder groups. These results indicated that infection with both strongyles species directed the effort of immunotransmitters to the production of antibodies as reflected by elevation of IL-4. On the other hand, this effort was diverted to both cellular response and antibodies production as indicated by equal balance. In the same way, low infection was unable to change the balance that already found in apparently healthy group.

Results in this study found that after strongyles infection Th1 as represented by TNF-α increased followed by an increase in Th2 as represented by IL-4. It could be suggested that the combined elevation of TNF-α and FEC with the decrease in serum antibodies might reflect early infection because early infection had no chance or time to stimulate B-cell to produce antibodies. However, high FEC with elevation in serum antibodies and IL-4 might confirm the incidence and progression of infection. Concerning TNF-α/IL-4 balance, low FEC with elevated serum antibodies and IL-4 could prove the presence of infection. On the other hand, low FEC with decreased of serum antibodies and elevation of TNF-αmight indicate infection with other parasites rather than the intended one, and in this case, full screen of other antigens was recommended. It was clarified that the protective immune response against helminths has been referred as Th2 immune response [27].

## Conclusion

Hence, we concluded that combination of detecting Th1/Th2 balance and IgG concentration is good tool for appropriate diagnosis of such infection. In addition, the use of CSS antigen can be considered a precised immunodiagnostic antigen that can be used in serological diagnosis of *S. vulgaris*. More longitudinal research should be done concerning Th1/Th2 balance and the cross-reactivity of *S. vulgaris* and *Cyathostomins* spp. at the base of serological and molecular investigation.

## Authors’ Contributions

FAMA designed the study and contributed to experiments performance, laboratory work analysis and data interpretation, manuscript preparation and corresponded the authorship. SHMH contributed to experiments performance and further assisted in the manuscript preparation. AHE shared in experiments performance, and HMA contributed in sample collection. All authors have read and approved the final manuscript.
